# Potential relationship between cuproptosis and sepsis-acquired weakness: an intermediate role for mitochondria

**DOI:** 10.3389/fphys.2025.1520669

**Published:** 2025-03-20

**Authors:** Luying Yang, Leiyu Xie, Min Li, Yanmei Miao, Jun Yang, Shaolin Chen, Xinglong Ma, Peng Xie

**Affiliations:** ^1^ Department of Critical Care Medicine of the Third Affiliated Hospital (The First People’s Hospital of Zunyi), Zunyi Medical University, Zunyi, China; ^2^ Department of Nursing of Affiliated Hospital, Zunyi Medical University, Zunyi, China; ^3^ Department of Critical Care Medicine, The Second Affiliated Hospital, Hengyang Medical School, University of South China, Hengyang, China

**Keywords:** sepsis-acquired weakness, cuproptosis, mitochondria, copper, cell death

## Abstract

Sepsis is defined as a life-threatening organ dysfunction caused by a dysregulated host response to infection. Skeletal muscle atrophy due to critical illness is a common phenomenon in the intensive care unit (ICU) and is referred to as ICU-acquired weakness (ICU-AW). The occurrence of ICU-AW in patients with sepsis is known as sepsis-acquired weakness (SAW). Furthermore, it is well known that maintaining normal muscle function closely relates to mitochondrial homeostasis. Once mitochondrial function is impaired, both muscle quality and function are affected. Copper plays a key role in mitochondrial homeostasis as a transition metal that regulates the function and stability of various enzymes. Copper is also involved in oxidation-reduction reactions, and intracellular copper overload causes oxidative stress and induces cell death. Previous studies have shown that excess intracellular copper induces cell death by targeting lipid-acylated proteins that regulate the mitochondrial tricarboxylic acid (TCA) cycle, which differs from the known canonical mechanisms of regulated cell death. Furthermore, inhibitors of cell death, such as apoptosis, necroptosis, pyroptosis and ferroptosis, are not effective in preventing copper-induced cell death. This new form of cell death has been termed “Cuproptosis”; however, the mechanism by which copper-induced cell death is involved in SAW remains unclear. In this paper, we review the possible relationship between cuproptosis and SAW. Cuproptosis may be involved in regulating the pathological mechanisms of SAW through mitochondria-related signaling pathways, mitochondria-related ferroptosis mechanisms, and mitochondria-related genes, and to provide new ideas for further investigations into the mechanism of SAW.

## 1 Background

Sepsis is a life-threatening organ dysfunction caused by a dysregulated host response to infection (Singer et al., 2016), and is one of the leading causes of death in the intensive care unit (ICU), with a mortality rate of 30%–50% (Chen et al., 2019). In critically ill patients, common symptoms include skeletal muscle weakness and muscle atrophy, characterized by flaccid, symmetrical weakness of the skeletal muscles of the extremities. Respiratory muscles may also be affected, through a syndrome known as ICU-acquired weakness (ICU-AW) (Klawitter et al., 2023). The incidence of ICU-AW ranges from 25% to 100%, with risk factors including sepsis, immobilization, hyperglycemia, glucocorticoids, neuromuscular blocking agents, and multi-organ failure (Zorowitz, 2016). ICU-AW is most common in persistently critically ill patients (Latronico, 2016), and up to 60%–100% of patients with sepsis can develop ICU-AW, known as sepsis-acquired weakness (SAW) (Mitobe et al., 2019; Bellaver et al., 2023). ICU-AW is reportedly an additional organ failure after severe sepsis and septic shock (Schefold et al., 2010), and that mitochondrial dysfunction in skeletal muscle and lymphocytes is a key trigger of SAW (Maestraggi et al., 2017).

Mitochondria are encapsulated by an outer and an inner phospholipid membrane that divides the organelle into a matrix and a membrane space (Kühlbrandt, 2015). Mitochondria are the “powerhouses” of the cell, providing adenosine triphosphate (ATP) to the organism through oxidative phosphorylation. This process involves a group of enzymes assembled in the mitochondrial electron transport chain to provide energy. Additionally, mitochondria are involved in calcium homeostasis, intracellular reactive oxygen species (ROS) production, intracellular signaling mediation, and regulation of apoptosis (Kanova and Kohout, 2022). Maintaining mitochondrial quality is critical for the functioning of the organism. Therefore, cells have evolved a series of mitochondrial quality maintenance mechanisms to protect normal mitochondrial physiological functions (Ng et al., 2021). However, when any of these factors causes an imbalance in mitochondrial homeostasis, a series of dysfunctions and diseases, such as SAW, can occur (Maestraggi et al., 2017). Mitochondria accumulate copper for assembling copper enzymes, such as cytochrome c oxidase and superoxide dismutase 1 (SOD1). Thus, copper plays an important role in mitochondrial function and signaling related to mitochondrial bioenergetics, kinetics, and autophagy, affecting cell fate through mitochondrial metabolic reprogramming (Ruiz et al., 2021).

The role of copper primarily affects mitochondrial function and metabolism. Copper deficiency reduces activity of the mitochondrial respiratory complex, lowering the metabolic level (Jensen et al., 2019). Conversely, excessive copper accumulation can lead to apoptosis or necrosis (Tarin et al., 2023). Copper overload causes oxidative stress due to excessive accumulation of ROS, resulting in mitochondrial dysfunction and cell death (Yang et al., 2019; Liu et al., 2020a; Kang et al., 2019). In 2022, Tsvetkov et al. demonstrated that excess intracellular copper ions target lipoylated proteins that regulate the mitochondrial TCA cycle, leading to cell death through a mechanism distinct from known forms of regulated cell death (RCD). Interestingly, canonical cell death inhibitors, such as apoptosis, necrosis, pyroptosis, and ferroptosis inhibitors, failed to prevent copper-induced cell death. This new form of cell death is termed “Cuproptosis” (Tsvetkov et al., 2022) and copper is involved in mitochondrial metabolism and plays an important role in maintaining the morphological and functional integrity of mitochondria (Ruiz et al., 2021). The proposition of cuproptosis has advanced the understanding of cell death mechanisms, focusing on its occurrence in mitochondria and its reliance on mitochondrial respiration (Tsvetkov et al., 2022). Previous studies have highlighted the significance of mitochondrial disorders in conditions such as sepsis and ICU-AW (Maestraggi et al., 2017; Li X. et al., 2023; Nedel et al., 2023). In this study, we aim to elucidate the mechanism of cuproptosis in SAW, exploring the relationships between mitochondria, cuproptosis, and SAW, and proposing new ideas for its prevention and treatment.

## 2 The role of copper in sepsis

Copper exhibits strong redox activity and protein binding capacity, serving as a crucial cofactor for key enzymes involved in mitochondrial aerobic respiration, superoxide dismutation, and other vital biological processes. It is an essential trace metal for all living organisms (Huang et al., 2024). Additionally, copper metabolism is closely linked with other trace elements. Copper deficiency can impair iron mobilization, resulting in secondary iron deficiency (Araya et al., 2007), and when copper levels are elevated they exacerbate ferroptosis (Xue et al., 2023). Alterations in trace elements and heavy metal levels have been linked to sepsis. Specifically, elevated serum copper levels in septic patients suggest a potential association between copper and sepsis (Huang et al., 2024).

During sepsis, a decrease in pH due to systemic or localized acidosis can cause the release of copper from cuprocyanin and other carrier proteins, leading to elevated levels of free copper (Carr et al., 2015). Free copper can participate in various biochemical pathways, including the inactivation of activated protein C (APC), stimulation of endothelial cells (ECs), and the production of ROS (Zhou et al., 2022; Bar-Or et al., 2002; He et al., 2020). APC has anticoagulant and anti-inflammatory effects in severe sepsis. APC is generated from inactive protein C through the activation by thrombin in association with platelet regulatory proteins (Annane et al., 2013). However, sepsis impairs the regulation of thrombomodulin by inflammatory cytokines, which hinders the conversion of protein C to APC. Recombinant human APC, on the other hand, reduces serum interleukin-6 and plasma D-dimer levels, thereby improving survival in sepsis patients (Bernard et al., 2001). ECs are the main target of inflammatory mediators in sepsis (Li Y. et al., 2021). ECs contribute to sepsis pathogenesis through the activation of intracellular inflammatory pathways mediated by nuclear factor kappa B (NF-κB) and mitogen-activated protein kinase (MAPK), which are dependent on Toll-like receptors (TLR) (Khakpour et al., 2015).

Furthermore, copper overload damages T- and B-lymphocytes, leading to immunosuppression. Copper also influences apoptosis-regulating molecules in immune organs like the thymus and spleen, thereby inducing apoptosis in immune cells (Mitra et al., 2012). In addition, copper overload can disrupt mitochondrial energy metabolism (Ruiz et al., 2021). Low ATP levels lead to hyperphosphorylation of 5′adenosine monophosphate-activated protein kinase (AMPK), which in turn reduces the activity of mammalian Target of Rapamycin (mTOR). This reduction impairs cellular autophagy, ultimately resulting in compromised protein synthesis in sepsis and damage to skeletal muscle (Shi et al., 2020; Liao et al., 2020). Although copper deficiency affects SOD1 synthesis leading to impaired neurotransmitter release, neuromuscular junction instability and reduced muscle strength in mice (Shi et al., 2014), high intracellular copper concentrations still lead to neurological dysfunction and muscle atrophy (Sakellariou et al., 2014), further impairing neuromuscular function in septic patients. Additionally, several studies have shown that copper induces oxidative stress in cells, leading to muscle atrophy and inhibiting skeletal muscle regeneration (Wang et al., 2018a; Zhao et al., 2018; Wang et al., 2018b), suggesting that copper may play a similar role in SAW.

Interestingly, mitochondria consistently play a central mediating role in copper-induced cell death and sepsis pathogenesis. Although some reports indicate that the mitochondrial respiratory capacity of peripheral blood immune cells in sepsis patients may be increased or unchanged (Sjövall et al., 2013; Merz et al., 2017), other studies have demonstrated that impaired mitochondrial respiration in immune cells, such as macrophages and leukocytes, reduces their energy supply. This reduction in energy exacerbates septic immune paralysis (Weiss et al., 2020; McBride et al., 2020). Mitochondrial oxidative stress modulates copper-induced ECs dysfunction and is involved in sepsis pathogenesis (Zhou et al., 2022; Wang M. et al., 2023; Huet et al., 2009). ROS induces the upregulation of NOD-like receptor family pyrin domain-containing 3 (NLRP3), caspase-1, interleukin-1β (IL-1β), and interleukin-18 (IL-18) in ECs. This upregulation leads to the assembly and activation of the NLRP3 inflammasome through the thioredoxin-interacting protein. Activation of the NLRP3 inflammasome promotes the maturation of IL-1β and IL-18 and facilitates the formation of the pore protein Gasdermin D (GSDMD), which subsequently triggers cellular pyroptosis via the classical inflammasome pathway (Zheng et al., 2022). In addition, ROS-induced DNA damage can lead to hyperactivation of poly-ADP-ribose-polymerase-1 and trigger parthanatos in ECs (Li S. et al., 2022). The role of mitochondrial oxidative stress in sepsis mechanisms has been extensively described (van der Slikke et al., 2021; Bertozzi et al., 2024). Copper, an essential metallic element, plays a crucial role in regulating mitochondrial ROS (Zhou et al., 2022). Thus, mitochondria are key mediators in the pathogenesis of sepsis regulated by copper.

One view is that copper levels are elevated in patients with sepsis (Zhang et al., 2023; Akkaş et al., 2020), and animal studies have shown that inhibition of copper levels attenuates caspase-1 activation and reduces lymphocyte death, which in turn increases survival in models of sepsis (Deigendesch et al., 2018). This suggests that elevated copper levels may promote sepsis progression. However, another view is that copper may have some therapeutic value. During septic infections, the body can enhance antimicrobial function by increasing the release of copper (Huang et al., 2024). Copper selenium nanoclusters can produce synergistic antimicrobial effects for the treatment of mice with sepsis by *in situ* sulphation of endogenous H_2_S, triggering ROS bursts and photothermal therapy (Gao et al., 2022). These two views appear contradictory. However, the role of copper is complex and its role at different stages in the pathogenesis of sepsis remain poorly understood. While the body can enhance antimicrobial action during septic infections by increasing the release of copper, copper toxicity results from an elevated copper level, which cannot be controlled by the copper-resistant genes (Rafati Rahimzadeh et al., 2024), resulting in excessive accumulation of intracellular copper and thus inducing different forms of cell death (Huang et al., 2024). Recent studies have shown that cuproptosis occurs in cardiomyocytes during sepsis, which induces cardiotoxicity (Yan et al., 2024); Under these circumstances, does copper-induced cell death play a role in SAW?

## 3 Copper and cell death

Many previous studies have shown that copper-induced cell death is closely associated with ROS and inflammation, which triggers different forms of cell death including apoptosis, necroptosis, pyroptosis and ferroptosis. For example, elevated ROS levels caused by copper overload disrupt mitochondrial membrane permeability, leading to a decrease in mitochondrial membrane potential, which in turn triggers the release of apoptotic proteins, ultimately leading to apoptosis in mouse cells (Liu et al., 2020a). Copper overload activates the ROS/NF-κB signaling pathway and induces impaired mitochondrial autophagy, ultimately leading to pyroptosis (Zhou et al., 2022). Similarly, recent studies have shown that copper exposure induces an inflammatory response and concomitantly induces apoptosis, necroptosis and pyroptosis in mice (Zhao et al., 2024). Ferroptosis is an iron-dependent cell death characterized by disruption of iron homeostasis and lipid peroxidation reactions (Gao et al., 2019). Generally, iron is the inducer of ferroptosis, but interestingly, it was found that elesclomol promotes the degradation of copper ion-transporting ATPase alpha polypeptide (ATP7A). In this case, co-treatment of elesclomol and copper leads to copper retention in mitochondria, and excess copper induces the Fenton reaction leading to accumulation of ROS and enhanced cellular oxidative stress, ultimately leading to ferroptosis (Gao et al., 2021). Furthermore, copper promotes ferroptosis by inducing autophagic degradation of glutathione peroxidase 4 (GPX4) (Xue et al., 2023). However, the different modes of cell death mentioned above have something in common with cuproptosis, in that they can both be caused by copper overload.

In recent years, the proposal of cuproptosis has attracted attention. Cuproptosis, a newly defined form of cell death, is mainly caused by the loss of iron-sulfur (Fe-S) cluster proteins and aggregation of mitochondrial lipoylated proteins induced by intracellular copper overaccumulation, which together lead to proteotoxic stress (Tang et al., 2022). Previous studies have found that copper can interact with proteins and cause protein aggregation, but have not focused on copper-induced cytotoxicity (Weibull et al., 2019). Furthermore, unlike apoptosis, necroptosis, pyroptosis and ferroptosis, cuproptosis occurred independently of ROS production (Li et al., 2023b). As mentioned above, cell death including apoptosis, necroptosis, pyroptosis and ferroptosis can be induced by copper. That would also suggest that cuproptosis interacts with various forms of cell death rather than being independent of them.

## 4 Copper and mitochondria

### 4.1 Copper metabolism and mitochondria

The body obtains copper from the diet, which is absorbed by the epithelial cells of the small intestine and transported through the portal vein to the liver, where it enters the bloodstream or is excreted through the bile (Baker et al., 2017). This process involves the uptake and export of copper ions by specific transporter proteins (Maung et al., 2021). Copper is chelated and stored by metallothionein (MT), and excess copper is transported and excreted by specific peptides. Major copper chaperones include the cytochrome c oxidase copper chaperone (COX17), the copper chaperone for SOD1 (CCS) (La et al., 2010). Intracellular copper ions enter the mitochondrial matrix via COX17 and play a relevant role. In turn, copper ions in the cytoplasm bind to chelators, such as glutathione (GSH) and MT, or are taken to SOD1, thereby regulating intracellular ROS levels and copper homeostasis (Xie et al., 2023).

Copper is a cofactor for important enzymes, but excessive accumulation can lead to cellular metabolic disorders and death (Lelièvre et al., 2020). Overloading or depleting metal ions within mitochondria can disrupt their morphology and function, causing cellular damage (Nam et al., 2018). Cytochrome c oxidase (COX) and SOD1 are copper-dependent enzymes (Garza et al., 2023). Copper deficiency hinders COX assembly, reduces ATP production, triggers oxidative stress and exacerbates cell death (Li F. et al., 2022). SOD is an antioxidant defence system and copper supplementation enhances its activity and maintains mitochondrial function. In conclusion, copper is closely related to mitochondrial function (Ruiz et al., 2021; Acetoze et al., 2017; Zeng et al., 2020; Zheng et al., 2023).

### 4.2 Copper-induced cell death and mitochondria

**FIGURE 1:**
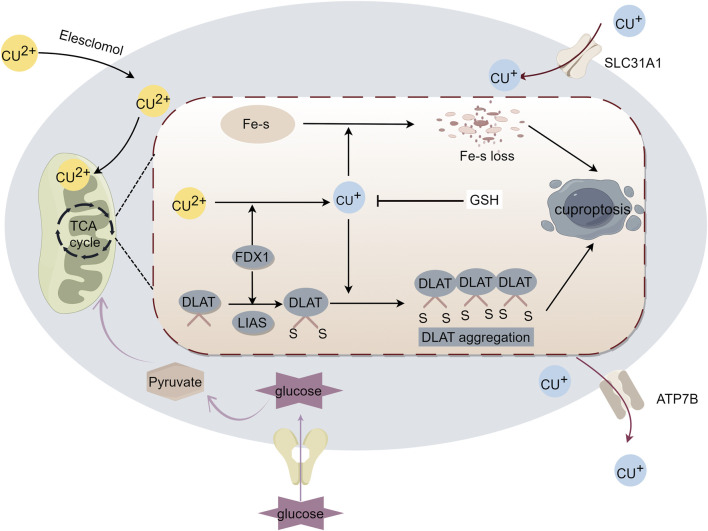
Mechanism of cuproptosis. Excess intracellular copper targets lipoylated proteins that regulate the mitochondrial tricarboxylic acid (TCA) cycle to induce cell death. Excess copper enters the cell, and ferredoxin 1 (FDX1) reduces copper to toxic monovalent copper ions, which results in the lipoylation and oligomerization of proteins involved in the TCA cycle, as well as iron-sulfur cluster protein loss. Together, these factors lead to proteotoxic stress and induce cuproptosis.

Although copper overload has been found to stimulate COX biosynthesis and assembly in the absence of cytotoxicity, resulting in the production of physiological amounts of ROS in the erythroid cell line K562, thereby improving mitochondrial function, copper has also been shown to improve mitochondrial function (Jensen et al., 2019; Ruiz et al., 2016). However, Tsvetkov et al. proposed that cuproptosis is induced by proteotoxic stress caused by an excess of intracellular copper ions. On the one hand, FDX1 encodes a reductase that enhances copper ion toxicity and promotes degradation of iron-sulfur cluster proteins. On the other hand, FDX1/LIAS is an upstream regulator of protein lipoylation and promotes the aggregation of lipoylated mitochondrial enzymes. Together, these aberrant processes trigger proteotoxic stress and cell death (Tsvetkov et al., 2022) (Figure 1). The above studies suggest that cuproptosis involves a copper-triggered mode of mitochondrial cell death, challenging the conventional view that oxidative stress is the underlying molecular mechanism of metal-induced toxicity, thus reinforcing the view that mitochondria are multifaceted regulators of cell death (Tang et al., 2022). Whether the mechanism involves the previously elucidated oxidative stress-induced cytotoxicity mechanism due to copper overload or the hypothesized copper-regulated cell death, it should be noted that mitochondria are always involved.

## 5 SAW and mitochondrial dysfunction

SAW is primarily characterized by skeletal muscle atrophy and weakness, often affecting skeletal and respiratory muscles, and in severe cases, it can lead to quadriplegia (Schefold et al., 2010). Although the pathophysiological mechanism of SAW remains incompletely understood, it is currently thought to be associated with skeletal muscle mitochondrial dysfunction (Maestraggi et al., 2017; Mankowski et al., 2021; Chatre et al., 2017). Damaged mitochondria fuse with healthy mitochondria to rescue impaired function, thereby preserving the overall physiological function of the mitochondrial network (Gustafsson and Dorn, 2019). However, in sepsis, healthy mitochondria decrease in the diaphragm muscle cells, leading to a reduced level of mitochondrial fusion marker mRNA and increased levels of non-functional small optic atrophy one protein isoforms. This inhibits mitochondrial fusion, resulting in dysfunction of the diaphragm (Wai et al., 2015; Oliveira et al., 2021). Reduction in mitochondria has been linked to an increased release of mitochondrial degradation products and damage-associated molecular patterns, which interact with their receptors, thereby stimulating the secretion of inflammatory factors in peripheral cells (Mankowski et al., 2021; Zhang et al., 2010). Conversely, the prolonged inflammatory process of sepsis increases tumor necrosis factor α production, leading to reduced mitochondrial respiration and impaired mitochondria biosynthesis, in mouse skeletal muscle (Mofarrahi et al., 2012). Additionally, mitochondrial respiratory complexes were dysfunctional and quantitatively defective in muscle samples from patients with sepsis compared to healthy controls, further confirming the association between mitochondrial dysfunction and SAW (Jiroutková et al., 2015). Tsvetkov et al. have demonstrated that mitochondria play a role in copper ion-overload-induced cell death (Tsvetkov et al., 2022). Thus, copper may regulate SAW by disrupting mitochondrial homeostasis.

## 6 Cuproptosis and SAW:intermediate role of mitochondria

The intracellular accumulation of copper ions is the key to copper-induced cell death, which is dependent on the regulation of mitochondrial respiration. Cells that are dependent on mitochondrial respiration are almost 1,000 times more sensitive to copper ions than cells dependent on glycolysis (Tsvetkov et al., 2022). Approximately two-thirds of the copper in the human body is found in bone and muscle, and skeletal muscle is rich in mitochondrial ions (Liu Y. et al., 2022). ATP produced by mitochondrial respiration provides energy for skeletal muscle movement (Hood et al., 2019). In addition, severe mitochondrial damage is observed in the skeletal muscle of patients with sepsis and multiple organ failure in the ICU, and the muscle ATP concentration is significantly reduced (Brealey et al., 2002). A study has confirmed that septic cardiotoxicity is associated with cuproptosis (Yan et al., 2024). Thus, cuproptosis may play a role in SAW through impaired mitochondrial function.

### 6.1 Copper may induce SAW through mitochondria-related signaling pathways

**FIGURE 2:**
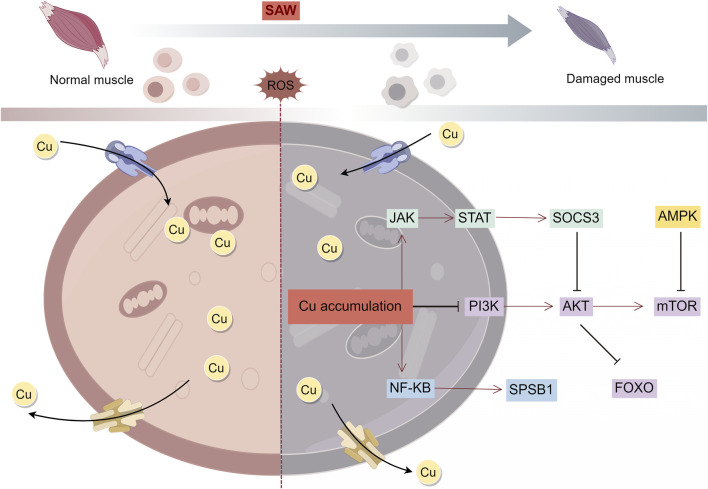
Mechanism of copper overload leading to SAW. Intracellular copper overload induces mitochondrial dysfunction and thus regulates SAW through multiple signaling pathways.

Serine/threonine kinase (AKT) is involved in mitochondria-mediated apoptosis, oxidation-reduction state, dynamic homeostasis, autophagy, and metabolism (Xie et al., 2022). Copper accumulation significantly reduces antioxidant enzyme activity, disrupts mitochondrial dynamics, and inhibits the phosphatidylinositol-3-kinase/AKT/mTOR (PI3K/AKT/mTOR) pathway in chicken skeletal muscle (Wang et al., 2018b). Notably, the PI3K/AKT/mTOR pathway is inhibited by copper accumulation, which induces septic skeletal muscle atrophy (Yin et al., 2022) and atrophy of the diaphragm (Wu et al., 2019). Members of the forkhead box O (FOXO) family are downstream targets of AKT (Farhan et al., 2017; Zhang and Zhang, 2019), and the FOXO pathway also play a role in SAW. For example, copper-induced cell death is associated with increased expression of FOXO1, FOXO3 and FOXO4 (Zeng et al., 2022; Hassani et al., 2018), which induces mitochondrial dysfunction by triggering mitochondrial dynamics and excessive autophagy (Chen et al., 2023). In addition, FOXO1 is an important factor in proteolysis, and increased FOXO1 expression during sepsis is a key factor that causes SAW (Smith et al., 2010; Castillero et al., 2013).This suggests that copper-induced cell death may be involved in the pathomechanism of SAW through the FOXO-related signaling pathway. Excess copper activates the Janus kinase/signal transducer and activator of transcription (JAK-STAT) pathway (Deng et al., 2023), and JAK-STAT activation induces mitochondrial autophagy and upregulates suppressor of cytokine signaling 3 (SOCS 3) expression, which in turn inhibits AKT expression through an insulin-dependent pathway, ultimately leading to SAW (Zanders et al., 2022; Huang et al., 2020). In addition, energy metabolism plays an important role in SAW (Jiang et al., 2022). NF-κB is involved in energy metabolism by regulating glycolytic utilization and mitochondrial respiratory homeostasis (Vander Heiden et al., 2009). Copper accumulation also activates the NF-κB pathway (Deng et al., 2023; Liu et al., 2020b), which induces an increase in SPRY domain-containing SOCS box protein 1 (SPSB1) and has been found to lead to muscle atrophy and weakness in mice with sepsis (Li et al., 2023c). AMPK is a critical cellular energy sensor that regulates cellular metabolism and maintains energy homeostasis (Herzig and Shaw, 2018). Under energy stress, AMPK enhances the mitochondrial ATP production pathway and reduces ATP consumption pathways, to reduce unnecessary energy consumption (Hardie, 2011). Therefore, AMPK activation is an adaptive mechanism in sepsis (Jin et al., 2020). Interestingly, excess copper activates the AMPK-mTOR signaling pathway, and activation of AMPK inhibits mTOR expression, leading to impaired mitochondrial function and ultimately cell death (Lei et al., 2021). Thus, copper may be involved in the activation of AMPK in sepsis and play a role in SAW. Therefore, copper may induce SAW through mitochondria-related pathways such as PI3K/AKT/mTOR, FOXO, JAK-STAT, NF-κB, AMPK-mTOR, and others (Figure 2).

### 6.2 Copper may induce SAW through mitochondria-associated ferroptosis

**FIGURE 3:**
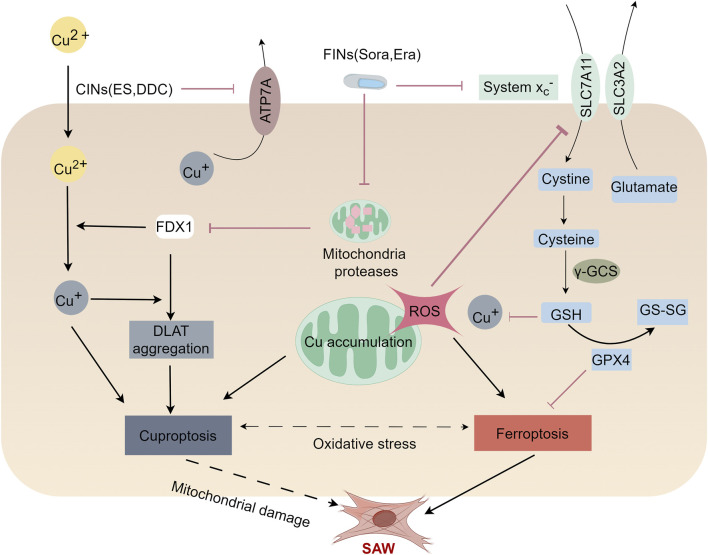
Mechanisms of cuproptosis and ferroptosis in the SAW. Ferroptosis and cuproptosis may crosstalk with each other and induce SAW by impairing mitochondrial function. Ferroptosis inducers (FINs), Sorafenib (Sora) and Erastin (Era) promote cuproptosis induced by copper ion carriers (CINS) by stabilizing the ferredoxin 1 (FDX1) protein and depleting the intracellular glutathione (GSH) level. Mechanistically, FIN stabilizes FDX1 by inhibiting mitochondrial proteases. Stabilized FDX1 enhances protein-lipid acylation and facilitates the transfer of the reduced copper ion, Cu^+^. In addition, FINs inhibit cystine input by inhibiting the Xc-system, which leads to a lower GSH level and thus a higher copper ion concentration. Together, these factors enhance the aggregation of lipoylated proteins, thereby promoting cellular cuproptosis. Elesclomol decreases the expression of ATP7A, resulting in intracellular retention of copper, which in turn leads to ROS accumulation. This effect promotes the degradation of SLC7A11, which further enhance oxidative stress and ultimately lead to ferroptosis. This suggests that ferroptosis and cuproptosis may crosstalk with each other and induce SAW by impairing mitochondrial function.

Ferroptosis, is a form of iron-dependent cell death that is induced by the overaccumulation of iron and lipid peroxides. Mitochondria, the central organelles responsible for iron metabolism and energy production, play a key role in ferroptosis (Gao et al., 2019). Moreover, cuproptosis also occurs within mitochondria, and the onset of cuproptosis is accompanied by disruption of mitochondrial morphology (Cobine and Brady, 2022). Mitochondrial respiration and mitochondrial enzymes play key roles in regulating cuproptosis (Tang et al., 2022).Several recent studies have demonstrated the existence of crosstalk between the mechanisms of cuproptosis and ferroptosis. Elesclomol as a copper ion carrier promotes the degradation of ATP7A. In this case, co-treatment of elesclomol and copper leads to copper retention in mitochondria, and excess copper induces the Fenton reaction, leading to accumulation of ROS and enhanced cellular oxidative stress, and ultimately to ferroptosis (Gao et al., 2021). Furthermore, ferroptosis inducers inhibit mitochondrial matrix-associated protease-mediated degradation of ferredoxin 1 (FDX1), which in turn upregulates protein lipidation and induces cuproptosis by reducing the intracellular synthesis of the copper chelator GSH via the inhibition of cystine production (Wang W. et al., 2023). Whereas mitochondria play a role in the crosstalk between cuproptosis and ferroptosis. Specifically, it is manifested in the mitochondrial TCA cycle. Studies have demonstrated the necessity of the TCA cycle and glutamine catabolism in ferroptosis, and blockade of the TCA cycle or glutamine deficiency ameliorates ferroptosis induced by cystine depletion or erastin (Gao et al., 2019). FDX1, a key factor in cuproptosis, promotes lipoylation of the mitochondrial enzyme DLAT thereby affecting the TCA cycle (Tang et al., 2022). In addition, GSH not only acts as a common inhibitor of ferroptosis and cuproptosis, but also participates in the regulation of the mitochondrial TCA cycle (Liu and Chen, 2024). The tumor suppressor p53 is an important metabolic regulator, p53 not only promotes mitochondrial TCA cycling but also plays an important role in cuproptosis (Zhang et al., 2023). In different cellular environments, p53 is thought to promote or inhibit ferroptosis (Akkaş et al., 2020; Deigendesch et al., 2018). The above studies have revealed that cuproptosis and ferroptosis have similarities, with strong associations between the mechanisms of cuproptosis and ferroptosis. Importantly, ferroptosis is involved in sepsis-induced muscle atrophy and weakness and that STAT6 inhibition significantly reduces mitochondrial dysfunction and thus rescues ferroptosis in the skeletal muscle of mice with sepsis (Sheng et al., 2024).This suggests that cuproptosis may induce SAW through the mitochondrial dysfunction mechanisms that have been associated with ferroptosis. This also suggests that copper homeostatic imbalance exacerbates ferroptosis, which in turn is involved in the induction of cuproptosis, which in turn leads to SAW (Figure 3).

### 6.3 Cuproptosis -related genes regulate SAW through mitochondria

A genome-wide Clustered Regularly Interspaced Palindromic Repeats (CRISPR)/CRISPR-associated protein nine screen revealed that cuproptosis was highly associated with 10 genes: *FDX1*, *LIPT1*, *LIAS*, *DLD*, *DLAT*, *PDHA1*, *PDHB*, *MTF1*, *GLS*, and *CDKN2A* (Tsvetkov et al., 2022).

Notably, *DLAT*, *DLD*, *PDHA1*, and *PDHB* are subunits of the pyruvate dehydrogenase complex (PDC) (Pavlu-Pereira et al., 2020). Mitochondrial PDC irreversibly decarboxylates pyruvate to acetyl coenzyme A, which feeds the mitochondrial TCA cycle (Stacpoole, 2017) and plays an important role in mitochondrial aerobic respiration (Park et al., 2018). Decreased PDC activity in the skeletal muscle of rats with sepsis leads to myocyte hypoxia and skeletal muscle dysfunction (Vary, 1996). Furthermore, *FDX1* is a key factor in copper-induced cell death and is involved in the formation of Fe-S cluster proteins, which are essential for mitochondrial function (Cai et al., 2017). Upregulation of *FDX1* leads to elevated cellular ROS and ferrous iron overload, which in turn induces ferroptosis, due to the continuous accumulation of lipid peroxides (Li L. et al., 2023). Importantly, ferroptosis has been shown to be involved in the development of SAW, suggesting that FDX1 may also be involved in the regulation of SAW (Sheng et al., 2024).

A bioinformatic analysis of the relationship between the pathogenesis of sepsis and cuproptosis-related genes identified *LIAS* and *PDHB* as potential diagnostic biomarkers for cuproptosis-associated sepsis (Sun et al., 2024). *LIAS* is responsible for encoding components in the lipoic acid pathway and for synthesizing the potent mitochondrial antioxidant α-lipoic acid (LA) (Krishnamoorthy et al., 2017). Furthermore, previous studies have shown that *LIAS* plays a key role in maintaining redox and mitochondrial homeostasis in sepsis (Sun et al., 2024). Cuproptosis-related genes are closely associated with sarcopenia and that the upregulation of *LIAS* gene expression promotes LA accumulation in mitochondria, thereby reducing oxidative damage in skeletal muscle (Lin et al., 2022). Therefore, increasing *LIAS* expression may rescue SAW by facilitating the maintenance of mitochondrial homeostasis.

Pyruvate Dehydrogenase E1 Subunit Beta (PDHB) protein is a key enzyme of the mitochondrial TCA cycle, which plays a crucial role in cellular energy metabolism by acting on acetyl coenzyme A and promoting its entry into the TCA cycle, ultimately producing ATP (Lee and Banerjee, 2020). Downregulation of *PDHB* is strongly associated with development of sepsis (Sun et al., 2024). *PDHB* is considered to be a protective factor for muscle tissue that promotes myogenic differentiation and improves muscle function by inhibiting the forkhead box P1-ariadne two homolog axis (Jiang et al., 2023). Therefore, targeting *PDHB*, a key gene for cuproptosis, may be an important means of treating SAW.

High mobility group protein B1 (HMGB1) is a pro-inflammatory factor that induces a lethal systemic inflammatory response in the late stages of sepsis, and targeting HMGB1 may reduce the impact of sepsis and the associated organ damage (Deng et al., 2022). HMGB1 induces mitochondrial oxidative damage (Dong et al., 2015), whereas HMGB1 inhibition activates the AMPK pathway and attenuates mitochondrial dysfunction (Liu et al., 2021). Copper accumulation-induced ATP depletion activates AMPK to promote HMGB1 phosphorylation, which leads to an increased extracellular release of HMGB1. HMGB one release precedes elesclomol-copper induced cell death, which suggests that HMGB1 may serve as a sensitive predictor of early cuproptosis (Liu J. et al., 2022). In addition, HMGB1 inhibition rescues muscle atrophy caused by cachexia (Li L. et al., 2021); therefore, cuproptosis through HMGB1 may be a key factor in sepsis-induced SAW.

In addition to mitochondria, relationships exist between copper and SAW. For example, copper directly binds to GPX4 proteins, leading to the formation of GPX4 aggregates and subsequent autophagic degradation of GPX4, which in turn induces ferroptosis (Xue et al., 2023). As discussed above, ferroptosis is involved in SAW (Sheng et al., 2024), and therefore, copper may be indirectly involved in SAW by directly inducing ferroptosis. GSH is a co-regulator of ferroptosis and cuproptosis and acts as an antioxidant in ferroptosis by hindering lipid peroxidation, thereby inhibiting cell death (Ursini and Maiorino, 2020). However, in cuproptosis, GSH acts as a copper ion chaperone to chelate copper ions, attenuates the aggregation of fatty acylated proteins, and inhibits cell death induced by excessive intracellular copper ions (Tsvetkov et al., 2022). Glutamine (Gln) depletion in skeletal muscle is significant in patients in the ICU after severe trauma and sepsis, and Gln supplementation is protective against sepsis-induced skeletal muscle injury (Hou et al., 2021). While Gln is a precursor of GSH, Gln deficiency causes a decrease in GSH concentration (Matés et al., 2020). As a key inhibitor of cuproptosis, a decrease in GSH concentration provides an opportunity for cuproptosis to occur. In addition, when there is severe Gln deficiency, muscle atrophy occurs (Cruzat et al., 2018), which further strengthens the correlation between cuproptosis and SAW. In addition, a recent study has shown that N-acetylcysteine (NAC) alleviates sepsis-induced muscle atrophy by downregulating endoplasmic reticulum stress (Chen et al., 2024). Interestingly, as a precursor of GSH, NAC functions not only to regulate redox homeostasis but also to chelate copper ions to alleviate copper toxicity (Wolfram et al., 2020) (Table 1).

**TABLE 1:** Copper-related genes in relation to mitochondria and SAW.

Copper-related genes	Relationship with mitochondria	Relationship with SAW	References
DLAT, DLD, PDHA1, PDHB	Encoding the mitochondrial PDC and interacting with the PDC to provide acetyl coenzyme A for the TCA cycle	PDC downregulation leads to hypoxia and skeletal muscle dysfunction in septic myocytes	Pavlu-Pereira et al. (2020), Stacpoole (2017), Park et al., 2018; Vary (1996)
FDX1	Involving in Fe-s cluster protein formation and regulation of mitochondrial ROS	Involving in iron death and indirectly regulates SAW	Wang et al. (2023b), Cai et al. (2017), Li et al. (2023d)
LIAS	Encodeing mitochondrial LA and regulating mitochondrial redox homeostasis	Regulation of redox balance in septic skeletal muscle	Sun et al. (2024), Krishnamoorthy et al. (2017), Lin et al. (2022)
PDHB	One of the key enzymes involved in the mitochondrial TCA cycle and in acetyl coenzyme A formation	PDHB downregulation is associated with sepsis development and PDHB promotes muscle differentiation to improve muscle function	Sun et al. (2024), Lee and Banerjee (2020), Jiang et al. (2023)
HMGB1	Induction of oxidative damage in mitochondria	Inhibition of HMGB1 treats sepsis and muscle atrophy	Deng et al. (2022), Dong et al. (2015), Liu et al. (2021), Liu et al. (2022b), Li et al. (2021b)
NAC	Regulation of mitochondrial redox homeostasis	Alleviation of sepsis-induced skeletal muscle atrophy	Chen et al. (2024), Wolfram et al. (2020)

Legends: DLAT: Dihydrolipoic amide S-acetyltransferase; DLD: Dihydrolipoic amide dehydrogenase; PDHA1: Pyruvate dehydrogenase E1 subunit alpha 1; PDHB: Pyruvate dehydrogenase E1 subunit β; FDX1: Ferredoxin 1; LIAS: Lipoic acid synthase; HMGB1: High mobility group protein B1; NAC: N-acetylcysteine.

Copper has been reported to have a partial bactericidal effect in the treatment of septicemia (Huang et al., 2024; Gao et al., 2022). However, it has also been claimed that copper induces interleukin-8 expression and plays a role in early inflammation in sepsis (Bar-Or et al., 2003). Furthermore, cuproptosis has been shown to be involved in septic myocardial injury (Yan et al., 2024), thus it is possible to speculate that sepsis promotes cuproptosis in skeletal muscle cells, inducing SAW.

## 7 Conclusion

The copper level is elevated in humans with sepsis, but animal studies suggest that copper may have some therapeutic effect. One possible explanation is that copper, although it has some antimicrobial properties, has a limited role in counteracting the systemic inflammatory response of sepsis, leading to a constant release of copper from the organism; however, when the copper level exceeds the threshold that the organism can tolerate, cytotoxicity is exacerbated, which can lead to cell death. This cell death may coincide with the onset of cuproptosis; after all, overloading the body with copper levels also provides an opportunity for cuproptosis to occur. And the mechanism of cuproptosis is likely to accompany the development of sepsis and SAW.

The role of copper in sepsis and cell death has been extensively studied, but its role in copper-induced cell death in SAW remains poorly understood. This paper reviews how copper overload-induced cuproptosis may contribute to SAW pathogenesis through mitochondria-related pathways, including signaling pathways (such as PI3K/AKT/mTOR, FOXO, JAK-STAT, NF-κB, AMPK-mTOR), cuproptosis-related genes (like DLAT, DLD, PDHA1, PDHB, FDX1, LIAS, HMGB1, NAC), and ferroptosis. As a newly identified mode of cell death, the regulatory pathways of cuproptosis in SAW require further investigation. Current studies on cuproptosis largely rely on bioinformatics, however, the specific regulatory mechanisms regarding cuproptosis in sepsis as well as in skeletal muscle cells are not known. More cellular and animal experiments are urgently needed to complement this to clarify its relationship with SAW.

Importantly, cell death does not occur through isolated pathways; rather, various cell death pathways interact to induce cell death at different stages of sepsis. Mitochondria play a crucial role in regulating cell death across multiple pathways. Future research should use mitochondria as a focus to explore the mechanisms of cuproptosis in SAW, particularly concerning mitochondrial oxidative stress, energy metabolism, and the TCA cycle. These insights could offer new targets for understanding SAW mechanisms and for the diagnosis and treatment of patients.

## Glossary

ICU: Intensive care unit
ICU-AW: Intensive care unit-acquired weakness
SAW: Sepsis-acquired weakness
ATP: Adenosine triphosphate
ROS: Reactive oxygen species
SOD1: Superoxide dismutase 1
TCA: Tricarboxylic acid
RCD: Regulated cell death
APC: Activated protein C
ECs: Endothelial cells
NF-κB: Nuclear Factor kappa B
MAPK: Mitogen-activated protein kinase
TLR: Toll-like receptors
AMPK: 5' adenosine monophosphate-activated protein kinase
mTOR: mammalian Target of Rapamycin
IL-1β: Interleukin-1β
IL-18: Interleukin-18
GSDMD: Gasdermin D
NLRP3: NOD-like receptor family pyrin domain-containing 3
AKT: Serine/threonine kinase
Nrf2: Nuclear factor erythroid 2-related factor 2
Keap1: Kelch-like epichlorohydrin-associated protein 1
HSP70: Heat shock protein-70
MT: Metallothionein
COX17: Cytochrome c oxidase copper chaperone
CCS: Copper chaperone for SOD1
GSH: Glutathione
COX: Cytochrome c oxidase
Bcl-2: B-cell lymphoma-2
NAC: N-acetylcysteine
Fe-S: Iron-sulfur
PI3K: Phosphatidylinositol-3-kinase
FOXO: Forkhead box O
JAK: Janus kinase
STAT: Signal Transducer and Activator of Transcription
SOCS3: Suppressor of Cytokine Signaling 3
SPSB1: SPRY domain-containing SOCS box protein 1
GPX 4: Glutathione peroxidase 4
FDX1: Ferredoxin 1
CRISPR: Clustered Regularly Interspaced Palindromic Repeats
PDC: Pyruvate dehydrogenase complex
LA: α-lipoic acid
PDHB: Pyruvate dehydrogenase E1 subunit β
HMGB1: High mobility group protein B1
Gln: Glutamine
ATP7A: Copper ion-transporting ATPase alpha polypeptide
